# Fall time may be a reliable discriminator between neoplastic and non‐neoplastic urinary bladder lesions in dogs undergoing contrast‐enhanced ultrasound: a pilot study

**DOI:** 10.1111/vru.13105

**Published:** 2022-06-09

**Authors:** Carlotta Spediacci, Martina Manfredi, Giulia Sala, Tiziana Liuti, Nicolas Israeliantz, Davide Danilo Zani, Mauro Di Giancamillo, Maurizio Longo

**Affiliations:** ^1^ Departement of Veterinary Medicine and Animal Science (DIVAS) University of Milan, Street of University n. 6 Lodi (LO) 26900 Italy; ^2^ Royal Dick School of Veterinary Studies University of Edinburgh Edinburgh Scotland UK

**Keywords:** Canine, Neoplasia, Polypoid cystitis, Bladder Disease, Quantitative CEUS

## Abstract

Contrast‐enhanced ultrasound (CEUS) can provide quantitative information on enhancement patterns and perfusion of lesions, based on time‐intensity curves (TICs). No published studies have compared CEUS parameters in neoplastic and non‐neoplastic urinary bladder lesions in dogs. The aim of the current prospective, pilot study was to quantitatively characterize the CEUS pattern of neoplastic and non‐neoplastic urinary bladder lesions in dogs, assessing the influence of contrast arrival time (CAT) on the final appearance of the curves. Fourteen dogs with cyto‐histopathological diagnoses were included (seven malignant and seven inflammatory lesions). B‐mode ultrasound was performed followed by CEUS examination after an intravenous bolus injection of 0.04 mL/kg of contrast medium, and TICs were elaborated by dedicated software. Receiver operating characteristic curves (ROC) for each TIC parameter were obtained. Neoplastic lesions had subjectively shorter rise time (RT), time to peak (TTP) and fall time (FT) than inflammatory lesions. Based on ROC curve analyses, fall time ≥ 10.49 s was the most reliable parameter for diagnosing non‐neoplastic disease in this small sample of dogs (area under the curve [AUC] 0.75, sensitivity 83.33%, specificity 66.67%). No difference was found between ROCs calculated for each parameter of TICs by adding or removing CAT. Results of the current study provide background for future, larger scale studies evaluating use of a CEUS FT threshold of 10.49 s as a possible discriminator for urinary bladder neoplastic lesions in dogs.

**List of abbreviations**
AUC: Area under the curve
CAT: Contrast arrival time
CEUS: Contrast‐enhanced ultrasound
CT: Computed Tomography
ECVDI: European College of Veterinary Diagnostic Imaging
FIG: Figure
FT: Fall Time
MHz: Megahertz
mTT: Mean transit time
ROC: Receiver operating Curves
ROI: Region of interest
RT: Rise time
s: Seconds
SI: Maximum signal intensity
SD: Standard deviation
TIC: Time‐intensity curve
TTP: Time to peak
UCC: Urothelial cell carcinoma
US: Ultrasound
WHWT: West Highland White Terrier

## 1 INTRODUCTION

Urinary bladder neoplasms account for approximately 1–2% of all malignant tumors in dogs.^1^ Epithelial tumors are the most common, representing approximately 97% of all malignant neoplasms, including urothelial cell carcinoma (UCC), followed by squamous cell carcinoma and adenocarcinoma.^2^ Other less common neoplasms of non‐epithelial origins are leiomyoma, leiomyosarcoma, fibroma, hemangioma, hemangiosarcoma, and lymphoma.^3^, ^4^, ^5^ The urinary bladder is also frequently affected by non‐neoplastic pathologies, such as urolithiases and cystitis.^6^ ^;^ ^7^ Common clinical signs include hematuria, stranguria, and other forms of dysuria. Additionally, in dogs affected by neoplastic conditions, lameness, weight loss, and lethargy are rarely reported.^5^ Diagnostic ultrasonography (US) is a standard diagnostic imaging test for dogs with suspected urinary bladder neoplasia. This is due to its ease of use, low cost, and excellent real‐time contrast resolution.^8^ Demhiwall et al. described thickening of the bladder wall as the most common US finding in inflammatory diseases of the urinary tract. Similar findings were also observed by other authors.^9^ ^;^ ^10^ Polypoid cystitis in particular, with mass‐like mucosal proliferation and severe diffuse bladder wall thickening, may present overlapping characteristics with urinary neoplasia. On US, UCC is characterized by an intramural infiltration with high vascularization, and most frequently affect the bladder trigone.^9^ ^;^ ^11^, ^12^, ^13^, ^14^ Urinary bladder polyps may show similar characteristics, with pedunculated mural masses often multifocal in distribution.^15^ ^;^ ^16^ Moreover, US shows low specificity in differentiating between benign and malignant bladder lesions.^17^ Definitive diagnosis for urinary neoplasms in dogs require invasive tests such as transcutaneous fine‐needle aspiration, cystotomy, cystoscopy‐guided biopsies, or traumatic catheterization. Furthermore, transcutaneous procedures are discouraged due to the risk of tumor seeding.^5^ ^;^ ^18^

A quantitative, ultrasonographic method for discriminating neoplastic versus non‐neoplastic urinary bladder masses would therefore be helpful for improving prognosis and treatment planning in affected patients. Previous studies in dogs have described the use of CEUS for the detection of splenic and hepatic malignancies in pets. However, both quantitative and qualitative analyses of CEUS of the lower urinary tract are poorly described in veterinary medicine, with a recent study focusing on UCC in a limited cohort of canine patients.^30^ The authors described a vivid enhancement of the neoplasms, with rapid wash‐in and a slower wash‐out phase, with loss of wall layering. In human medicine, the extent of angiogenesis in malignant neoplasms of the urinary bladder has been reported to be associated with tumor growth and metastasis formation and CEUS is considered useful for the diagnosis of urinary bladder neoplasia.^19^, ^20^, ^21^, ^22^, ^23^ The differentiation of neoplastic and non‐neoplastic lesions of urinary bladder might be performed quantifying the CEUS pattern derived from time‐intensity curves (TICs).^27^ A thorough evaluation of both the urinary bladder layers and tumoral angiogenesis is essential to establish the degree of aggressiveness of the urinary bladder neoplasms and thus to define an accurate prognosis.^39^ The close analogy between canine and human urothelial cell carcinoma has been demonstrated in numerous studies, making the dog an animal model for studying this disease.^40^

The use of CEUS may represent an important ancillary technique in daily practice, particularly in the case of challenging bioptic procedures. These contrast agents consist of gas microbubbles encapsulated by a shell of different compositions.^23^ The gas core makes the microbubbles highly echogenic such that each bubble can be ultrasonographically detectable.^24^ Most US contrast agents do not diffuse across the endothelium and therefore remain strictly within the vasculature and microvasculature, allowing accurate assessment of vascular perfusion.^1^, ^2^, ^3^, ^4^, ^5^, ^6^, ^7^, ^8^, ^9^, ^10^, ^11^, ^12^, ^13^, ^14^, ^15^, ^16^, ^17^, ^18^, ^19^, ^20^, ^21^, ^22^, ^23^, ^24^, ^25^, ^26^, ^27^, ^28^ Following this, the gas content of the contrast agent is eliminated through the lungs, which represents a safe route of clearance, with short‐time adverse events occurring in only 0.2% of dogs and cats.^25^, ^26^ The CEUS TIC is a quantitative analysis made using perfusion software, which analyzes the temporal sequence of images by measuring the change in pixel intensity in the region of interest (ROI). The signal intensity of each pixel over time is evaluated within the ROI. The final result of this process is a Gaussian curve that quantitatively describes the wash‐in and wash‐out phases of enhancement.^2^, ^3^, ^4^ ^;^ ^19^ ^;^ ^29^ ^;^ ^31^ The most important quantitative parameters to be considered are the time to peak (TTP), which measures the time from contrast injection to maximum signal intensity (SI); rise time (RT), from the increase of contrast enhancement to SI; Fall Time (FT), indicating the time that the signal takes to return from the peak enhancement to the baseline level; and mean transit time (mTT), which is defined as the total flow time of the contrast agent in the selected tissue (VueBox Quantification Toolbox, Instruction for use, Copyright 2019 Bracco Suisse SA).^21^;^29^, ^30^, ^31^ Another important parameter is the contrast arrival time (CAT), which indicates the time before the appearance of the first microbubble in the selected ROI. The CAT, which represents the first part of the curve, might be automatically removed by the software.^29^

Based on our review of the literature, there were no published studies comparing quantitative CEUS perfusion parameters between neoplastic and non‐neoplastic lesions of the urinary bladder in dogs. This study therefore aimed to quantitatively characterize neoplastic and non‐neoplastic lesions of the urinary bladder in dogs on CEUS, assessing the influence of CAT on the final appearance of the curves. We firstly hypothesized that CEUS TIC measures previously used for humans with urinary bladder neoplasia would be feasible for use in dogs and that a cut‐off value for some of these measures could be identified for predicting neoplasia versus non‐neoplasia in dogs. The second hypothesis was that CAT would represent a highly variable parameter between canine patients of different body conformation, and that this might influence the TIC when not excluded from analyses.

## 2 MATERIAL AND METHODS

### 2.1 Experimental design and subject selection criteria

This was a prospective pilot study. Procedures were approved by the Veterinary Ethics and Welfare Committee of the Royal School of Veterinary Studies of the University of Edinburgh (VERC approval 131.17). Informed owner consent was also obtained before enrollment of the dogs in the study. Dogs presenting to the Hospital for Small Animals at the University of Edinburgh between March 2018 and September 2019 for further investigation of lower urinary tract signs with urinary bladder changes visible on US were considered. Cases with ultrasonographic diagnosis of urinary bladder mural lesion in which a definitive diagnosis was reached based on histopathology, cytopathology, or microbiology were included. Dogs were excluded from the study if there was evidence of underlying heart disease or if excessive stress was induced by the procedure. All decisions regarding participant inclusion or exclusion criteria were made by an ECVDI‐certified veterinary radiologist (TL). The following clinical data were recorded for each dog by a third‐year ECVDI resident (ML): breed, sex, age, weight, and clinical signs. Dogs were divided into three categories according to weight: small (1–10 kg), medium (11–30 kg), and large (>30 kg).

### 2.2 Image acquisition techniques

All CT examinations were performed by the ECVDI third‐year resident (ML) under the supervision of the ECVDI‐certified veterinary radiologist (TL). Both were aware of signalment and clinical signs of the patients. All dogs also underwent a standardized B‐mode US examination of the lower urinary tract (Esaote MyLab Twice, Genova, Italy) using multi‐frequency (10–19 MHz) linear (LA435) and micro convex (SC3123) electronic array probes. All dogs were sedated and placed in right lateral recumbency. A small area in the caudal abdomen was clipped to avoid artifacts originating from the hair‐coat. The probe was placed in the long axis just cranial to the pelvic inlet and perpendicular to the skin within the ventral midline in females and on the side of the prepuce in males. A layer of gel was applied between the probe and the skin of the patient to obtain good contact with the transducer. The focal point was placed on the urinary bladder wall. For each examination, the presence or absence of lesions, distribution, echotexture, bladder wall thickening, presence of urolithiasis, or urinary sediment were recorded.

Following the B‐mode study, a contrast‐enhanced ultrasound (CEUS) examination was performed using a contrast‐tuned imaging module (CnTI^TM^, Contrast Tuned Imaging Technology). CEUS was performed using an electronic array probe with a contrast agent capability (SC3123). The lowest gain was set in order to highlight the contrast within the ROI.

A low mechanical index was used and selective placement of the focal zones to maximize the harmonic signal while minimizing the destruction of the contrast media were performed. The mechanical index was 0.3, and only one focal zone was analyzed, which was placed on the urinary bladder wall. The position of the patient and operators were not modified.

The contrast agent (sulfur‐hexafluoride echo‐signal enhancer, SonoVue®, Bracco Imaging, Milan, Italy) was administered manually in a rapid single bolus at a dosage of 0.04 mL/kg via injection into a direct access port connected to the IV catheter (20–22 G) placed in the cephalic vein. Each bolus was followed by a flush consisting of 2 mL of saline solution (0.9%).

The occurrence of adverse events was evaluated and recorded by a single observer (NI). Potential systemic side effects were monitored during sedation; cardiovascular and pulmonary parameters were monitored and reported in the anaesthesia report; any unpredictable changes were reported.

### 2.3 Image analyses

After administration of CEUS, for each dog a qualitative evaluation was performed by assessing the type of enhancement, that was defined as mild, moderate or marked and homogeneous or heterogeneous (Fig. 6), in agreement among the three observers who were blinded to final diagnosis (ML, TL, NI).

During each examination, a 2‐min digital video clip was recorded from the time of contrast injection. All raw data were stored in a local picture archiving and communication system and subsequently analyzed by three blinded observers (ML, TL, NI). Results with the highest Quality of Fit of the curves for each patient were recorded. Post‐processing quantitative analysis of the video clips was performed using image‐analysis software (Vue Box®, Bracco Imaging, Milan, Italy). For each dog, a ROI was drawn at the center of the lesion of interest. The ROI was drawn individually for each patient, selecting the smaller size and the most representative shape to avoid the inclusion of non‐representative peripheral tissues (Fig. 7).

Furthermore, for each ROI, a TIC perfusion model was elaborated by extrapolating the following data: SI, TTP, RT mTT, and FT. Each parameter was plotted in an Excel file sheet; afterward, each value was added to the CAT and also reported in the Excel file (Microsoft Excel 365, 2020 16.43 [20110804]).

### 2.4 Statistical Analyses

All statistical analyses were performed by a clinical statistician (GS) using dedicated software (SPSS 26.0, Mac IBM, Armonk, USA). Because of the small sample, data were analyzed by using descriptive rather than inferential statistics. Descriptive statistics were produced, and continuous variables were expressed as mean ± standard deviation (SD), while categorical variables were expressed as frequencies and percentages with 95% confidence intervals.

Power analysis was performed with the G‐power software using an alpha‐error of 0.05 and a sample effect of 0.5.

Receiver operating characteristic (ROC) curve was performed for each CEUS parameter (SI, TTP, RT mTT, and FT with and without CAT). The ROC was built to establish the optimal cut‐off value associated with neoplastic or non‐neoplastic lesions. The optimal cut‐off point was chosen using the Youden index, where sensitivity and specificity were maximized, and equal weight was given to false‐positive and false‐negative results. The calculated cut‐off values were used to calculate sensitivity and specificity. Additionally, the area under the curve (AUC) and 95% confidence intervals (CI) were calculated and used as indicators of the accuracy of the parameters.^33^ Interpretation of AUC was based on the following scoring system: 1.0 perfect test, 0.99–0.90 excellent test, 0.89–0.80 good test, 0.79–0.70 fair test, 0.69–0.51 poor test, and 0.50 or lower fail.^33^

## 3 RESULTS

Fourteen canine patients of different sexes, weights, and breeds met the inclusion criteria. Ten were female and four were male; the mean weight was 15.3 ± 9.4 kg, and the mean age was 8.5 ± 3.5 years. Two dogs were classified as large, three as small, and nine as medium size. Represented breeds were one of the following: cattle dogs, Chinese crested, Labrador Retriever, Lakeland Terrier, mixed breed, Norfolk Terrier, Podenco Canario, Schnauzer, Scottish Terrier, West Highland White Terrier, Weimaraner, Border Collie, and two Cocker Spaniels. The recorded clinical signs were hematuria, pollakiuria, and stranguria, and nocturia, and urinary incontinence in one case. In the neoplastic group, the definitive diagnosis was always obtained on the basis of a positive cytological examination performed by traumatic catheterization. In the non‐neoplastic group, diagnosis was performed by cystoscopy guided biopsy in one case (polypoid cystitis), traumatic catheterization in one case (dysplasia of epithelial cell), and in five cases the final diagnosis was made on the basis of cystocentesis and microbiological cultural examination.

Inflammatory lesions not cytologically‐histologically confirmed were clinically monitored with a complete resolution of the clinical signs and a normal one month follow up ultrasound.

Histological and cytological analysis revealed seven neoplastic lesions (UCC) and seven inflammatory diseases (one dysplasia of epithelial cell, one polypoid cystitis, four bacterial cystitis, and one cystolithiasis). In both the neoplastic and non‐neoplastic groups, five dogs were female and two were male. B‐mode US findings of neoplastic lesions were irregular thickening of the bladder wall with loss of normal layering and pedunculated round‐shaped masses, sometimes mineralized, located at the level of the urinary bladder trigone. US B‐mode of dogs with non‐neoplastic conditions showed generalized thickening of the bladder wall, polypoid masses, and in one case, hydroureter, hydronephrosis, and cystolithiasis were also detected. In all cases, the normal portion of the urinary bladder wall was identified as two parallel hyperechoic thin layers and a hypoechoic interposed layer that corresponded to the muscular layer.

**FIGURE 1:**
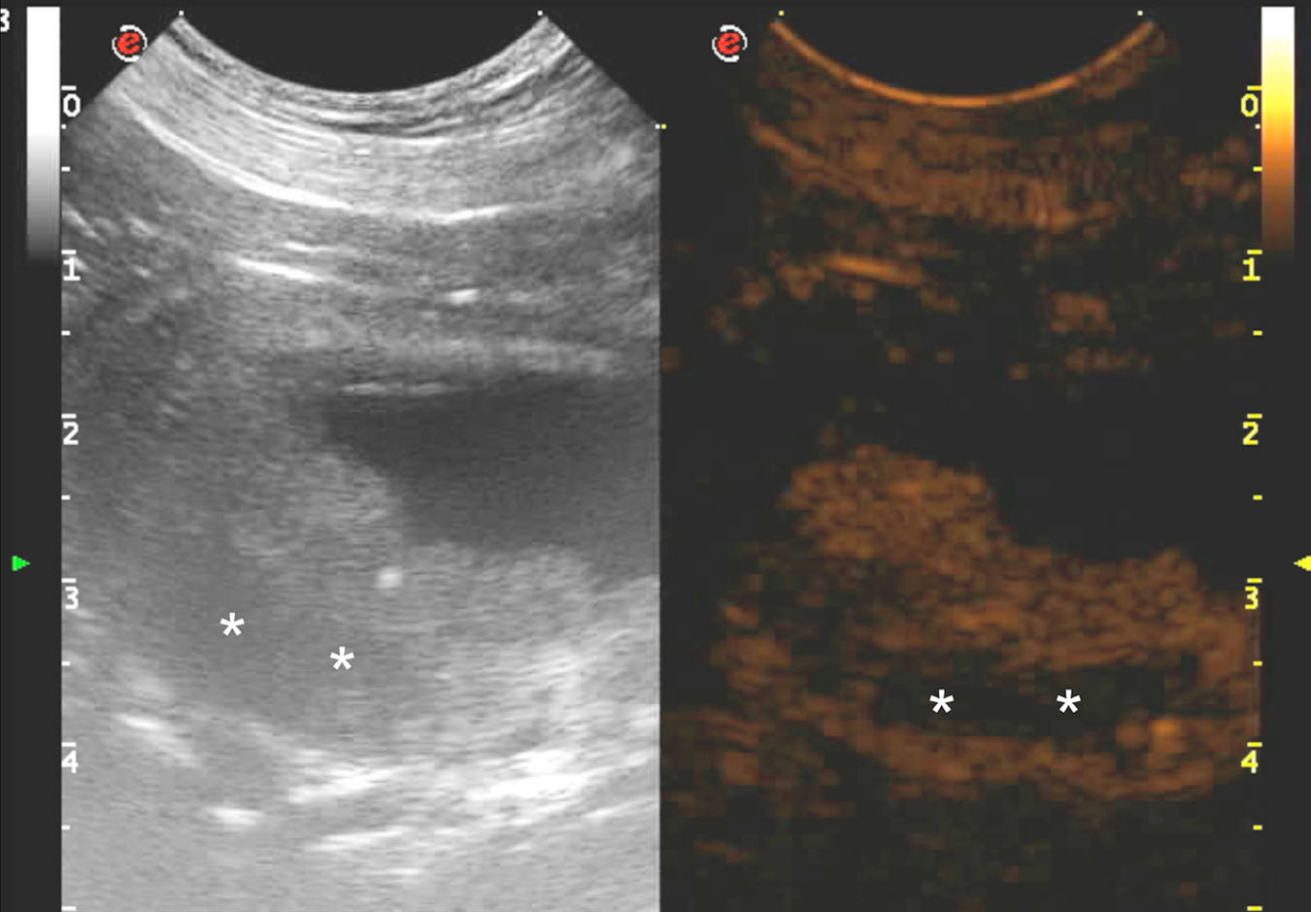
Contrast‐enhanced ultrasound images obtained with a multi‐frequency (10‐19 MHz) microconvex electronic array probe with a mechanical index of 0.3 from a dog with a neoplastic urinary bladder lesion. A: Long axis standard B‐mode sonogram of the urinary bladder showing a large inhomogeneous and poorly marginated mass occupying most of the lumen with multifocal hypoechoic areas (*). B: Long axis contrast‐enhanced ultrasound (CEUS) sonogram of the urinary bladder of the same lesion showing marked and heterogeneous enhancement [Colour figure can be viewed at wileyonlinelibrary.com]

**FIGURE 2:**
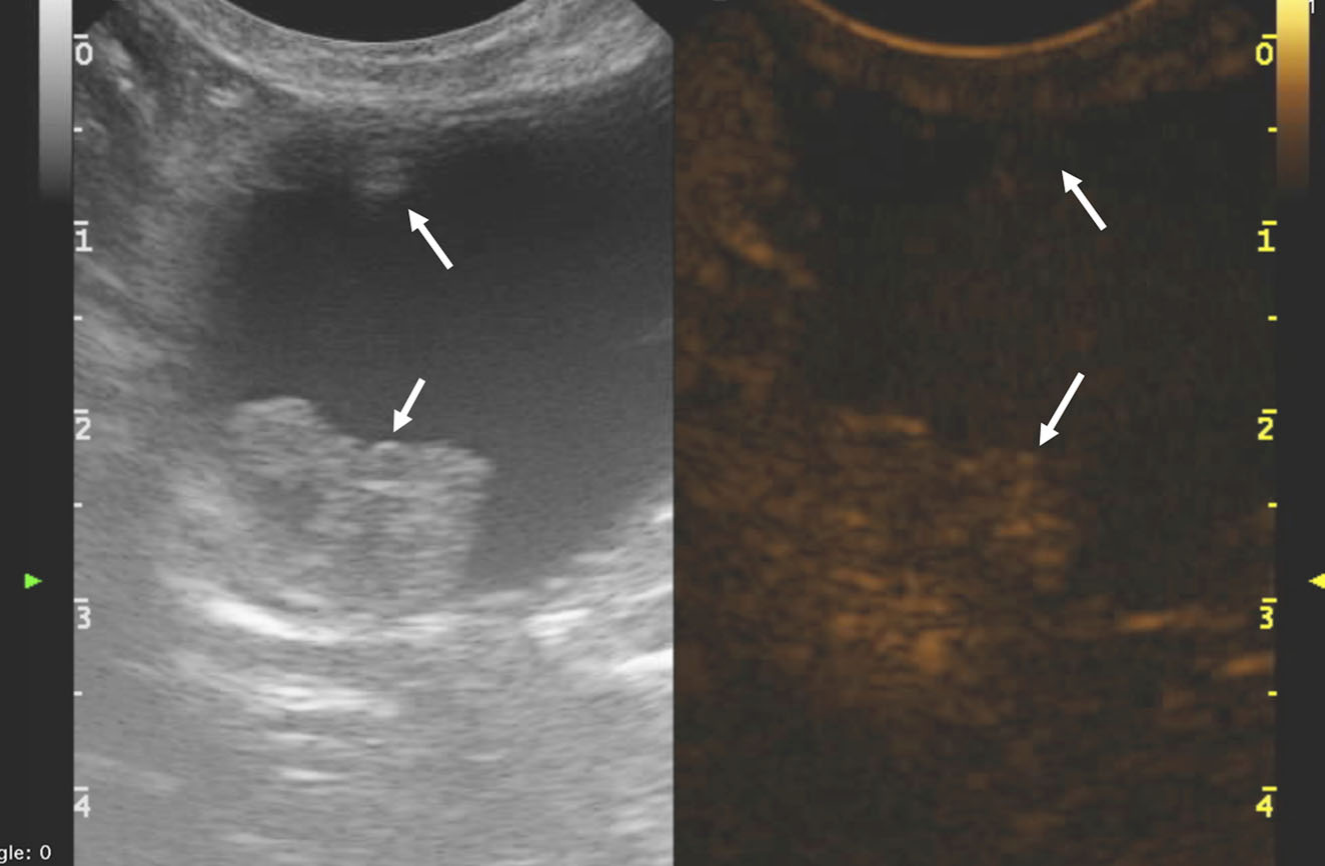
Contrast enhanced ultrasound images obtained with a multi‐frequency (10‐19 MHz) microconvex electronic array probe with a mechanical index of 0.3 from a dog with a non‐neoplastic urinary bladder lesion. A: Long‐axis standard B‐mode sonogram of urinary bladder showing two pedunculated lesions (arrows) extending into the bladder lumen, confirmed to be polypoid cystitis. B: Long‐axis contrast‐enhanced ultrasound (CEUS) sonogram of the urinary bladder of the same lesion showing moderate and homogenous enhancement [Colour figure can be viewed at wileyonlinelibrary.com]

Qualitative analysis of CEUS in neoplastic lesions showed moderate (4 of 7) to marked (3 of 7) and heterogeneous enhancement (7 of 7), with the presence of multiple non‐enhancing central areas likely compatible with necrosis. In two dogs of the neoplastic group only irregular thickening of the wall bladder was visible (Fig. 1). In these cases, assessing the CEUS pattern was challenging, given the ill‐defined margins of the lesion. In non‐neoplastic lesions, a mild (4 of 7) to moderate (1 of 7) and homogeneous (5 of 7) CEUS pattern was visible, with two cases showing mild thickening of the urinary bladder wall and absent enhancement (Fig. 2).

Contrast arrival time was highly variable between individuals, ranging between 2–29.5 s. The mean value of CAT in small dogs was 8 s, in medium‐sized dogs was 10.16 s, and in large dogs was 17.3 s (Table 3).

**FIGURE 3:**
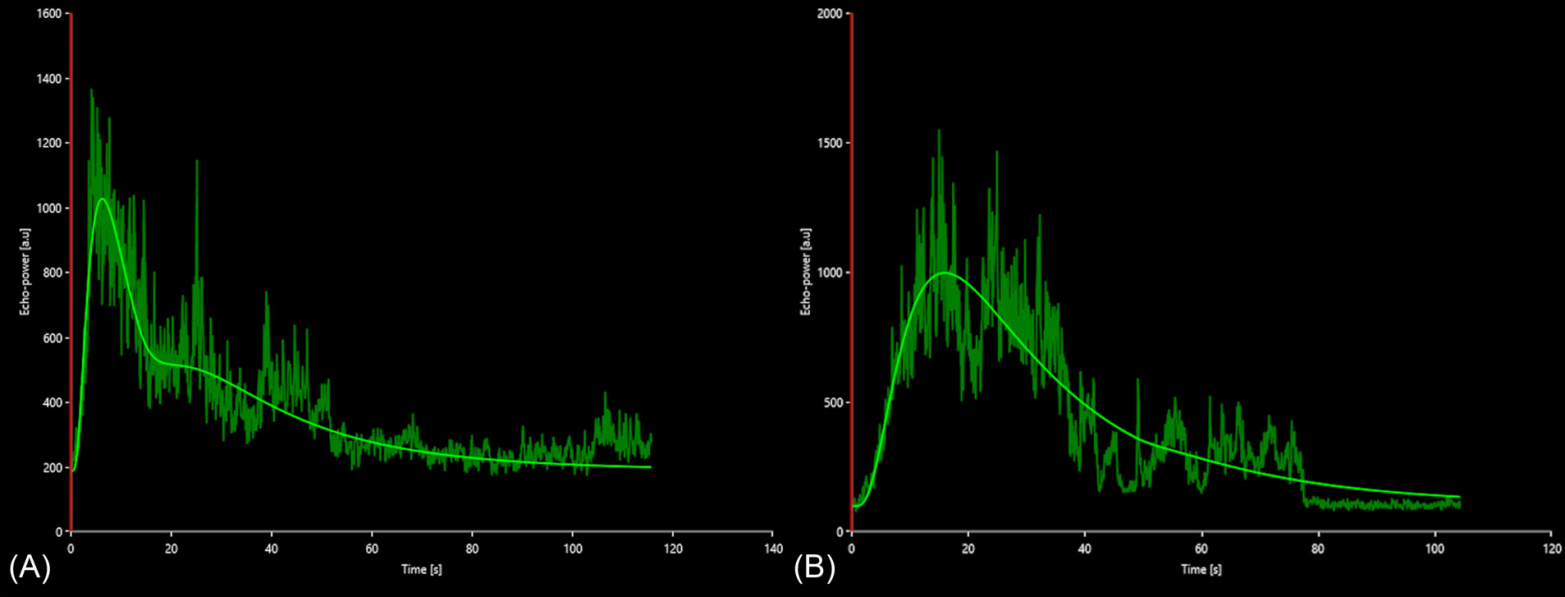
Quantitative CEUS analyses performed with VueBox. TIC is created from the ROI positioned in the lesions of two representative dogs, with a neoplastic‐lesion (A) and a non‐neoplastic lesion (B). Neoplastic lesion (A) shows a subjectively shorter TTP and RT, higher SI, and a more rapid FT compared with non‐neoplastic disease (B) [Colour figure can be viewed at wileyonlinelibrary.com]

Statistical comparison between the groups was not performed due to small sample sizes, but descriptive analyses of the quantitative parameters displayed neoplastic lesions with subjectively shorter RT, TTP, FT and longer mTT compared to inflammatory lesions (Fig. 3) (Table 1). From the analysis of the ROC curves (Table 2), FT ≥ 10.49 s proved to be the most accurate parameter in diagnosing non‐neoplastic disease (AUC 0.75, sensitivity 83.33%, specificity 66.67%). Moreover, RT ≥ 6.75 s and TTP ≥ 9.94 s were both indicative of a non‐neoplastic etiology (Fig. 5) (AUC 0.595, sensitivity 66.67%, specificity 71.43%). The AUC tested for the remaining parameters failed to differentiate between neoplastic and non‐neoplastic lesions (Table 2). No difference was found between the ROC analysis for each parameter by adding or removing CAT. Only the values without CAT were considered as relevant and reported in table 1 and 2. No immediate or delayed adverse reaction was detected during the examination.

**TABLE 1:** Statistical descriptive analysis of each contrast enhanced ultrasonographic  parameter for neoplastic and non‐neoplastic urinary bladder conditions in 14 dogs

Neoplastic Lesion (n = 7)					
	SI (a.u)	RT‐s	mTT‐s	TTP‐s	FT‐s
Mean	431.4	7.2	86.7	9.6	10.7
SD	781.7	6.4	114.4	7.3	4.3
Median	144.5	4.9	34.1	7.3	10.1
P25	21.4	3.6	25.4	4.8	8.9
P75	332.0	9.8	88.5	15.7	11.9
Non‐neoplastic Lesion (n = 7)					
	SI (a.u.)	RT‐s	mTT‐s	TTP‐s	FT‐s
Mean	118.8267	9.023333	65.48333	12.41667	22.245
SD	126.1924	5.908227	55.48983	7.449429	18.47987
Median	79.9	8.225	44.925	12.5	16.635
P25	19.09	4.9	24.49	7.08	10.49
P75	174.39	14.1	89.64	20.18	31.62

Abbreviation: a.u., arbitrary unit; SI, signal intensity; RT, rise time; mTT, mean transit time; s, seconds; TTP, time to peak; FT, fall time; SD, standard deviation

**TABLE 2:** Summary results of receiver operating curve analyses for each contrast enhanced ultrasonographic parameter in 14 dogs with neoplastic and non‐neoplastic urinary bladder disease

	Best cut‐off	Sensitivity	Specificity	Classified	AUC
SI‐a.u.	≥ 53.85 s	16.67%	85.71%	53.85%	0.333
RT‐s	≥ 6.75	66.67%	71.43%	62.23%	0.595
mTT‐s	≥ 40.83	66.67%	57.14%	61.54%	0.523
TTP‐s	≥ 9.94	66.67%	71.43%	69.23%	0.595
FT‐s	≥ 10.49	83.33%	66.67%	75.00%	0.750
	Best cut‐off	Sensitivity	Specificity	Classified	AUC
PE‐r	≥ 53.85 s	16.67%	85.71%	53.85%	0.333
RT‐s	≥ 6.75	66.67%	71.43%	62.23%	0.595
mTT‐s	≥ 40.83	66.67%	57.14%	61.54%	0.523
TTP‐s	≥ 9.94	66.67%	71.43%	69.23%	0.595
FT‐s	≥ 10.49	83.33%	66.67%	75.00%	0.750
RT‐s+CAT	≥ 18.9	66.67%	71.43%	69.23%	0.642
mTT‐s+CAT	≥ 45.63	66.67%	57.14%	61.54%	0.547
TTP‐s+CAT	≥ 25.2	66.67%	71.43%	69.23%	0.619
FT‐s+CAT	≥ 29.5	66.67%	83.33%	75%	0.722

Abbreviation: AUC, area under the curve; a.u., arbitrary unit; RSI, signal intensity; RT, rise time; mTT, mean transit time; s, seconds; TTP, time to peak; FT, fall time

**TABLE 3:** Statistical descriptive analysis of contrast arrival time for each dog size group

Size	Mean CAT	SD	Median	P25	P75
Large (n = 2)	17.3	13.8	17.3	7.5	27.1
Medium (n = 9)	10.2	8.5	8.05	5.05	11.8
Small (n = 3)	8	6.4	4.8	3.8	15.4

Abbreviations: CAT, contrast arrival time; SD, standard deviation; P25 _________, P75 __________

## 4 DISCUSSION

The quantitative CEUS parameters of neoplastic and non‐neoplastic lesions of the urinary bladder in canine patients were investigated. Among the analyzed parameters, only FT was found to be potentially useful for distinguishing neoplastic and non‐neoplastic lesions of the urinary bladder. No other parameters were distinctive for neoplastic and non‐neoplastic lesions of the urinary bladder. The second hypothesis related to the influence of CAT in the evaluation of TICs was not supported.

The ROC analyses demonstrated that FT was the most sensitive and specific parameter of the TIC in distinguishing neoplastic and non‐neoplastic conditions, with 10.49 s as a cut‐off to discriminate between the two groups. As shown in Table 1, the FT averages of neoplastic and non‐neoplastic groups differed (10.6 s and 22.24 s, respectively).

**FIGURE 4:**
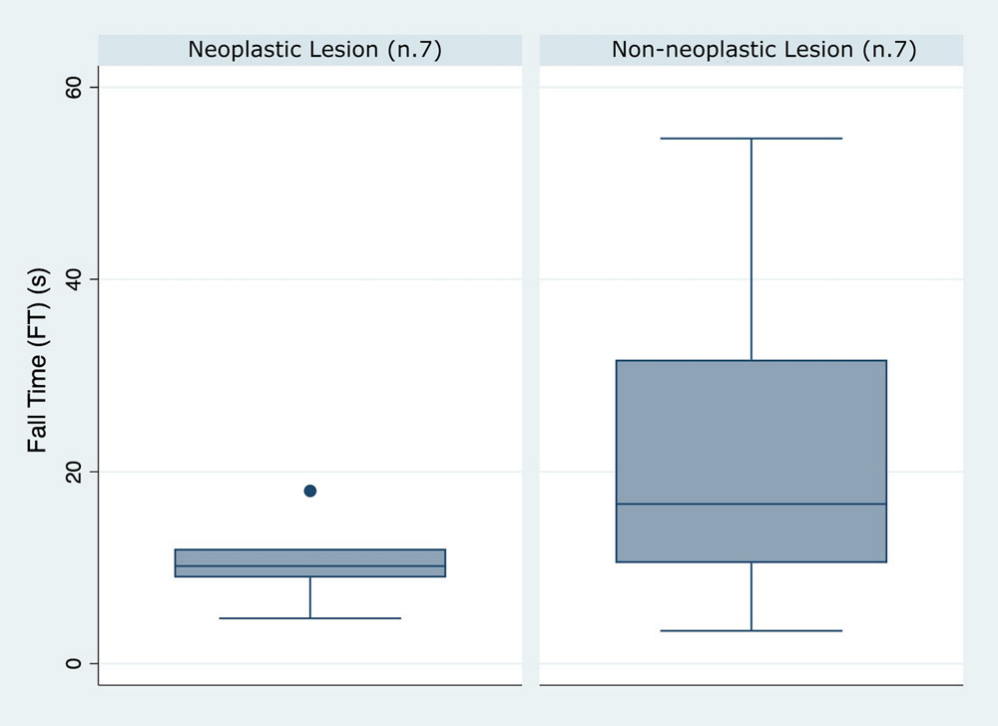
Box plot showing the distribution of Fall Time values in patients belonging to the neoplastic (n = 7) and non‐neoplastic (n = 7) group [Colour figure can be viewed at wileyonlinelibrary.com]

In the box plot the distribution of both groups is wide, particularly in the non‐neoplastic group (Fig. 4). Moreover, results are not normally distributed, with one outlier in the neoplastic group, which can be expected in a pilot study with a small sample size. Additionally, the ROI selection was challenging in cases without evidence of a well‐defined protruding mass, potentially altering the results, such as in patients with non‐polypoid cystitis (Fig. 2). A sensitivity and specificity of 75% has a limited usefulness in a clinical setting. However, median values of the two groups were different, suggesting that this value may potentially be of interest in a study with a larger population.

**FIGURE 5:**
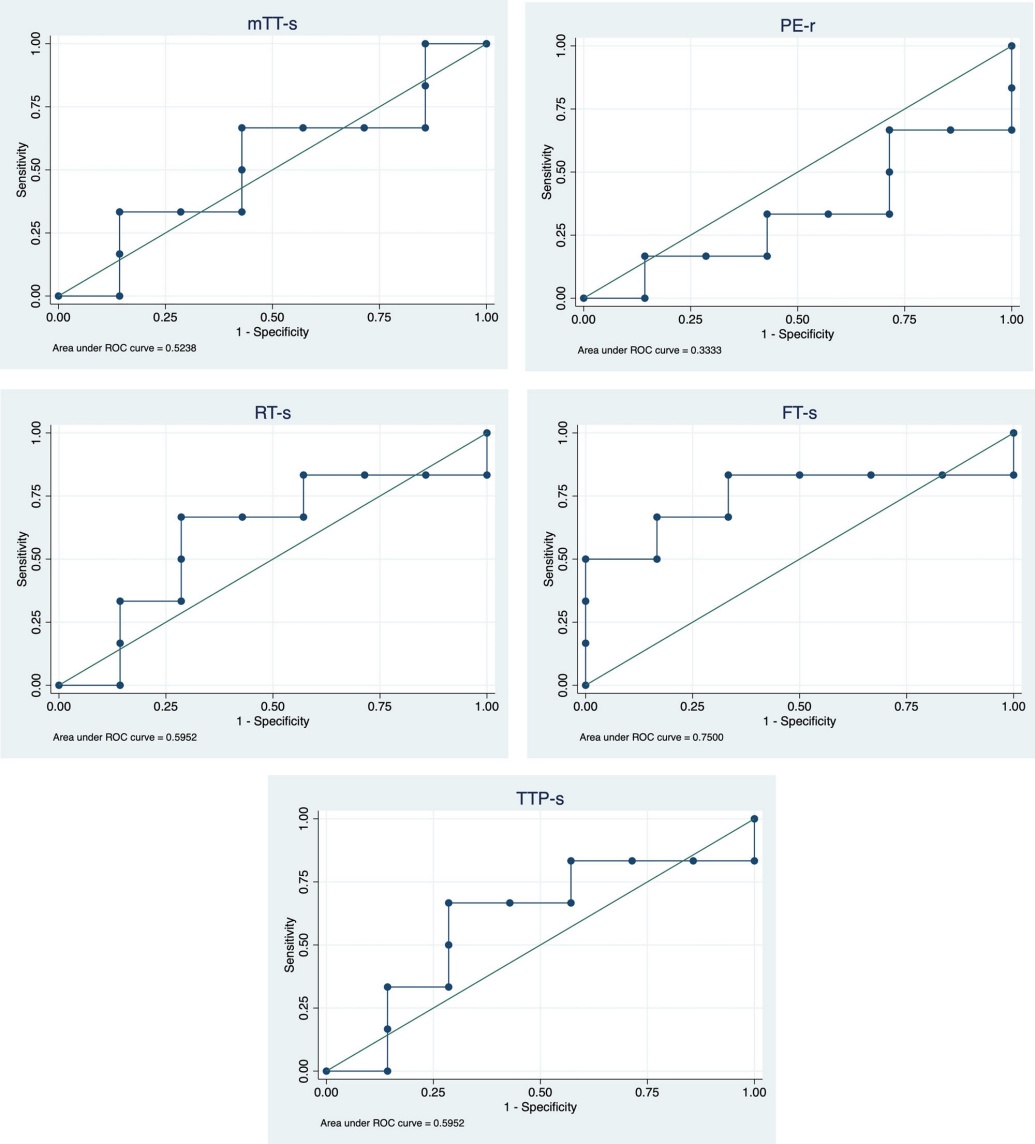
ROC curves of mTT‐s, PE‐r, RT‐s, FT‐s, TTP‐s [Colour figure can be viewed at wileyonlinelibrary.com]

**FIGURE 6:**
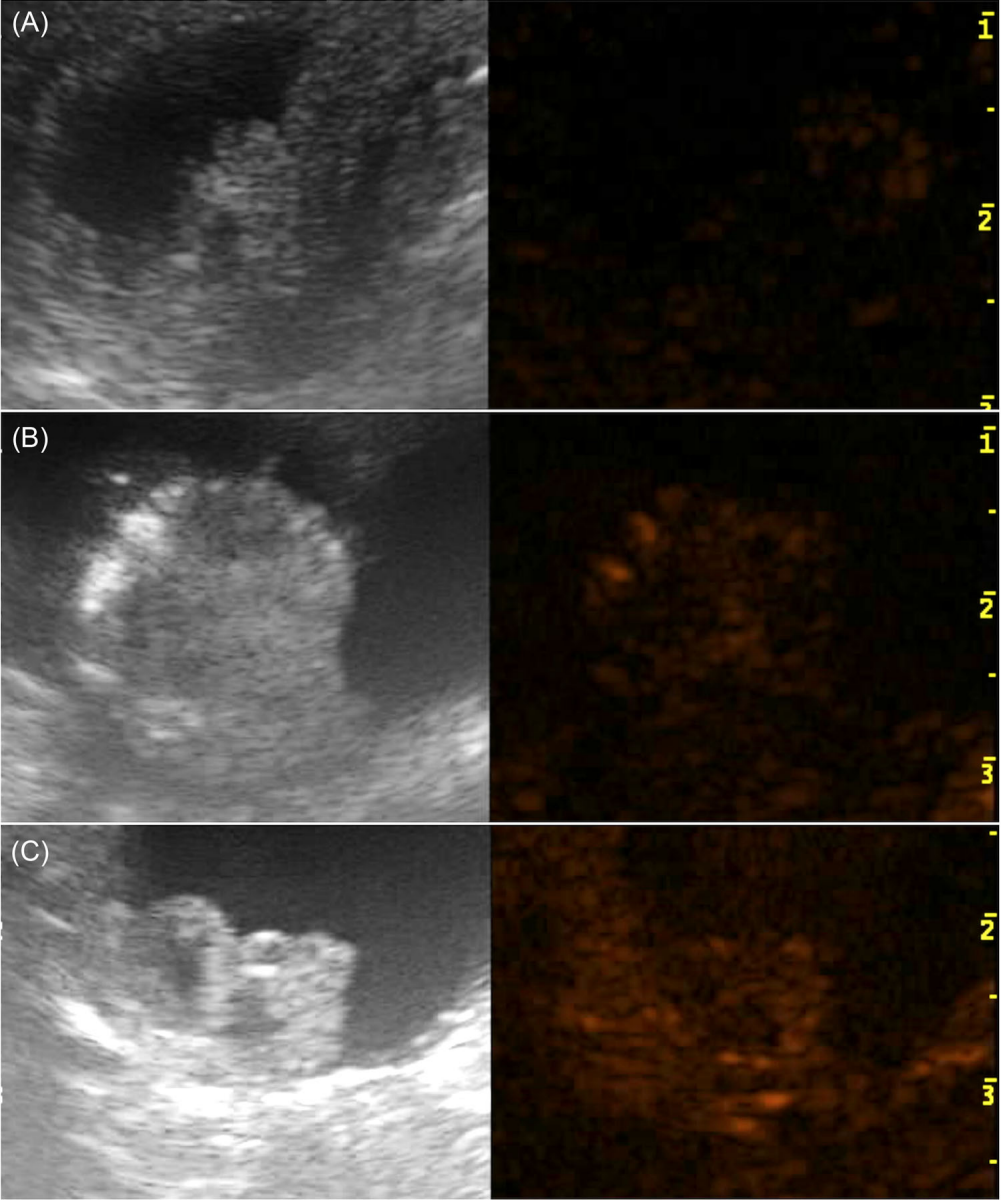
Contrast‐enhanced US images obtained with a multi‐frequency (10‐19 MHz) micro convex electronic array probes with a mechanical index of 0.3 after the injection of Sonovue. Images display three different urinary bladder lesion at the peak of contrast enhancement showing mild homogeneous enhancement (A), moderate heterogeneous enhancement (B), marked heterogeneous enhancement (C). Final diagnosis revealed inflammatory cystitis (A), urothelial cell carcinoma (B, C) [Colour figure can be viewed at wileyonlinelibrary.com]

**FIGURE 7:**
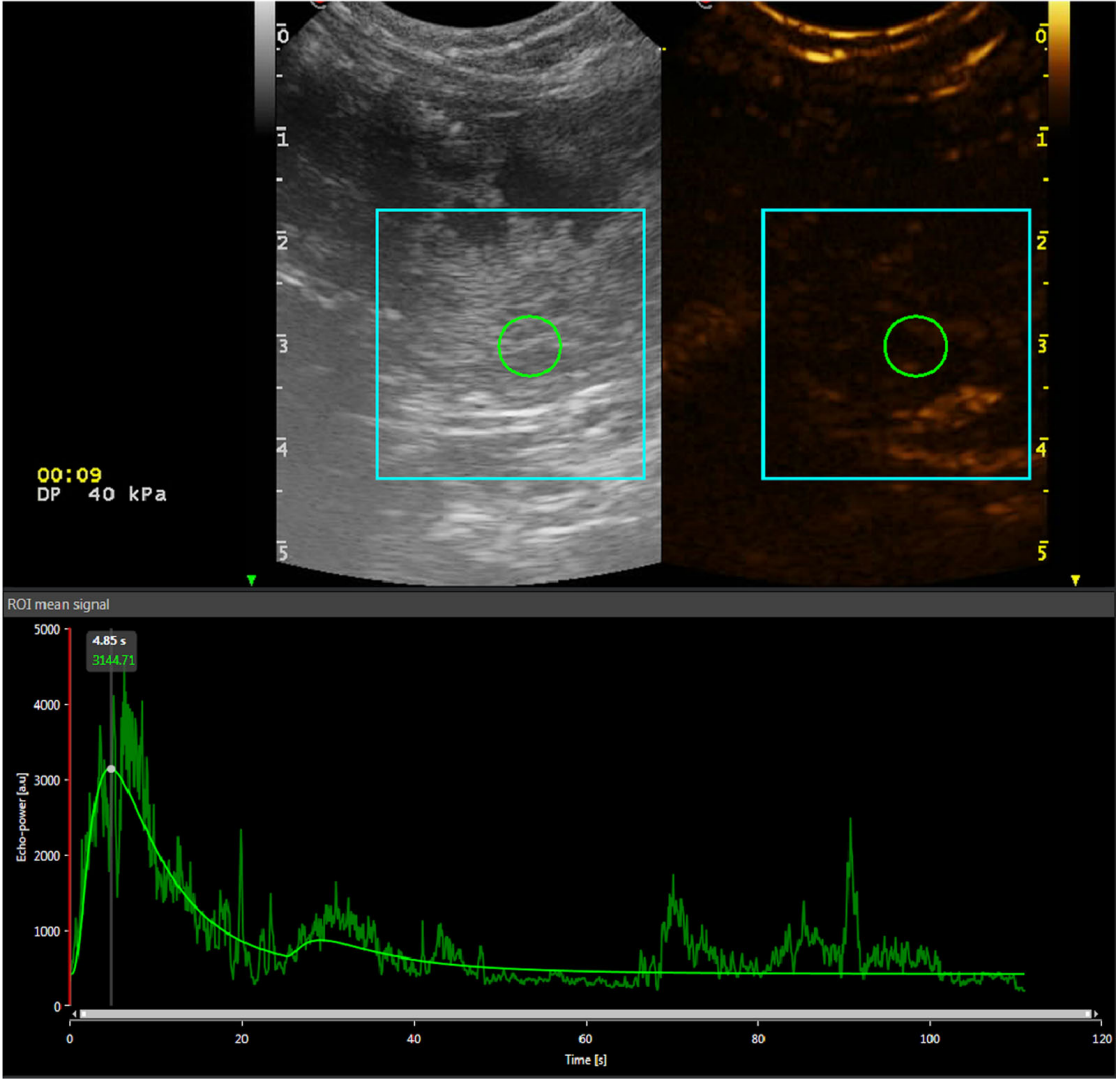
Placement of the region of interest (ROI) within the parenchyma of an urothelial cell carcinoma for quantitative analysis of the mass enhancement using Vue Box commercial software. The ROI is placed within the mass, maintaining distance from areas of necrosis and lesion margins to avoid including peripheral tissues within the ROI. A time intensity curve is generated from the ROI [Colour figure can be viewed at wileyonlinelibrary.com]

The CAT parameter was highly variable between individuals of different sizes. However, analysis of the ROC curves did not reveal a decrease in the sensitivity and specificity of any of the TIC parameters, including or removing the CAT value. This may be related to the over‐representation of medium‐sized dogs in our sample (9/14). The advantage of the software that does not take CAT into account when processing TICs may not have been detected in this study; however, it might be more evident in a population with a larger representation of toy/small and large/giant breeds, with a significantly longer or shorter CAT due to the related anatomical differences influencing contrast medium kinematics.

In veterinary medicine, few studies have evaluated the use of quantitative CEUS TICs for urinary bladder lesions. Pollard et al. reported that CEUS is a feasible technique for the evaluation of the lower urinary tract in dogs: in the cited study, a different contrast agent was used. However, no correlation between CEUS findings, vascular endothelial growth factor concentration, and criteria for assessing response to chemotherapy was found.^32^ Another recently published study, which aimed to describe the use of both qualitative and quantitative CEUS of UCC, reported that qualitative perfusion pattern analysis is more reliable than quantitative parameters to reach a definitive diagnosis as the US enhancement measurement might be influenced by many variables, such as cardiac output, blood pressure, and respiratory rate.^30^

In our study qualitative analysis of CEUS pattern showed a moderate to marked heterogeneous enhancement of the neoplastic lesion, in accordance with previous reports.^30^ A heterogeneous CEUS pattern is therefore more indicative of neoplastic infiltration while contrast intensity is overlapping between neoplastic and inflammatory lesions. In this group of dogs, the TIC shape of neoplastic and non‐neoplastic lesions was different. The development of easily accessible techniques that allow objective quantification of vascular patterns, such as quantitative CEUS, might be beneficial for the early differentiation of neoplastic and inflammatory lesions of the urinary bladder.

The small sample size of this study limited our ability to evaluate predisposition of breed, sex, and age or to compare findings with the previous literature. However, in accordance with previous studies, UCC appeared to be the most common in our sample, as it represented all tumors included in the study.^29^, ^30^, ^31^ Additionally, dogs with neoplastic diseases included predisposed breeds, such as West Highland White Terrier, Scottish Terrier, and Cocker Spaniel.^17^ ^;^ ^22^ ^;^ ^34^ ^;^ ^35^, ^36^ Transurethral cystoscopy biopsy could be considered the preferred diagnostic method in females; however, in male dogs, this method has been reported to be accurate in only 65% of cases.^37^ Urine cytology can lead to numerous false‐positive and false‐negative results, while percutaneous biopsies have been shown to increase the risk of needle‐track implantation; therefore, many authors recommend traumatic urethral catheterization to obtain a cytologic diagnosis of UCC.^5^ ^;^ ^15^ ^;^ ^17^ ^;^ ^38^

The first limitation of this study is the small sample size, which did not allow us to reliably define cut‐offs for each TIC parameter. Indeed, the power analysis with our sample was 14%, which is limited for a comparison between groups. The choice of selecting ROIs based on bladder lesions appearance meant that different depths were used, and this may have affected the SI, altering the results. Additionally, in cases of thin urinary bladder mural lesions the ROI selection proved to be particularly challenging. Furthermore, the sample was not heterogeneous in terms of patient size; therefore, it was not possible to adequately investigate the influence of CAT on the accuracy of the curves. Another limitation is the failure to consider heart rate and blood pressure, which could have provided interesting information about TIC variability. Additionally, the group of malignant neoplasia included only UCC; therefore, TICs might vary with other types of tumors.

In conclusion, the results of this pilot study suggest that neoplastic and non‐neoplastic diseases may present different TICs. A FT higher than 10.49 s may be the only reliable cut‐off to help characterizing neoplastic and non‐neoplastic lesions of the urinary bladder in dogs, although this result must be interpreted with caution. A combination of laboratory findings, standard B‐mode ultrasound and qualitative and quantitative CEUS analysis is still needed for further evaluation along with cytopathological confirmation. However, the results of this study need to be tested and statistically analyzed on a larger sample since the proposed cut‐off provides only limited information on the nature of UB lesions on CEUS.

## 5 LIST OF AUTHOR CONTRIBUTION

Category 1

6

a) Conception and Design: Spediacci, Liuti, Longo

b) Acquisition of Data: Israeliantz, Liuti, Longo

c) Analysis and Interpretation of Data: Spediacci, Manfredi, Sala, Longo, Zani, Di Giancamillo

Category 2

a) Drafting the Article: Spediacci, Manfredi, Sala

b) Revising Article for Intellectual Content: Spediacci, Manfredi, Liuti, Israeliantz, Longo, Sala, Zani, Di Giancamillo

Category 3

a) Final Approval of the Completed Article: Spediacci, Manfredi, Liuti, Israeliantz, Longo, Sala, Zani, Di Giancamillo

Category 4

a) Agreement to be accountable for all aspects of the work in ensuring that questions related to the accuracy or integrity of any part of the work are appropriately investigated and resolved: Spediacci, Manfredi, Liuti, Israeliantz, Longo, Sala, Zani, Di Giancamillo

## CONFLICT OF INTEREST

the authors declare that there are no financial interests related to article content.
